# Clinical characteristics and endoscopic treatment of hematospermia with postcoital hematuria

**DOI:** 10.1186/s12894-020-00646-x

**Published:** 2020-06-29

**Authors:** Zao-Ming Huang, Yan-Feng Li, Qi Wang, Yong Zhang, Yong Luo, Zhi-Lin Nie, Ke Li, Qing-Xing Feng, Xu-Dong Liu

**Affiliations:** 1 Department of Urology, Daping Hospital, Army Medical University, No.10, Daping Changjiangzhilu, Yuzhong District, Chongqing, 400042 China; 2 Department of Urology, Chongqing Renji Hospital, University of Chinese Academy of Sciences, Chongqing, 400062 China

**Keywords:** Posterior urethral hemangioma (PUH), Hematuria, Hematospermia, Urethrocystoscopy, Electroresection

## Abstract

**Background:**

Recurrent hematospermia accompanied by postejaculatory hematuria is a very rare phenomenon, has not been well understood in the clinical setting, and usually leads to misdiagnosis and mistreatment. The aim of this study was to summarize the clinical characteristics, etiologic diagnosis, and endoscopic treatment of hematospermia with postcoital hematuria.

**Methods:**

We collected the clinical data from 39 patients of hematospermia with postcoital hematuria, who were admitted to our hospital from May 2014 to October 2019. The etiologic diagnostic process and endoscopic surgery were analyzed retrospectively, and we observed and evaluated the efficacy and any complications during follow-up.

**Results:**

The average age of the 39 patients was 44.1 years (range, 18–61 years), and the disease history ranged from 1 month to 20 years, with a median duration of 24 months. All of the patients were observed by urethrocystoscopy, which showed 38 cases of posterior urethral hemangioma (PUH) or abnormal varicose vessels, and 1 case of anterior urethral hemangioma. Of these, 18 patients underwent transurethral resection of urethral hemangioma, and 21 patients underwent transurethral electrocauterization. Postoperative follow-up ranged from 1 to 56 months, with a median of 16 months. The symptoms disappeared in 37 patients and recurred in 2 patients two to 3 months after the operation. The two recurrent patients were treated again by transurethral electrocauterization, and their symptoms then disappeared.

**Conclusions:**

PUH is the most common cause of hematospermia with postejaculatory hematuria. Herein, we demonstrated that transurethral resection or electrocauterization provides a safe, effective, and minimally invasive method for the treatment of PUH.

## Background

Hematospermia accompanied by postejaculatory or posterection hematuria is a very rare clinical symptom in male patients. Although studies in recent years have shown that this phenomenon is primarily caused by posterior urethral hemangioma (PUH) [1–5], it is still easy to misdiagnose and mistreat due to its atypical clinical manifestations and an overall lack of relevant knowledge in this area [6]. According to our knowledge, until the end of 2019, the total number of reported male urethral hemangiomas in the MEDLINE database was less than 70 since the first case report in 1895 [1–21]. The clinical features, diagnosis, and treatment of this disease is still not well understood. From May 2014 to October 2019, 39 patients with intractable hematospermia accompanied by postejaculatory hematuria were treated at our hospital, and we herein summarize the clinical characteristics, etiologic diagnosis, and endoscopic treatment effects.

## Methods

Thirty-nine patients of intractable hematospermia with postcoital hematuria were treated at our hospital from May 2014 to October 2019. The clinical data were collected and retrospectively analyzed, including age, course of disease, principal symptoms, routine urine test results, coagulation function, imaging features, endoscopic findings, causes of bleeding, location and size of lesions, surgical operative skills, postoperative pathologic results, complications, and follow-up results.

All patients received conservative treatment for more than 1 month prior to admission, but no improvement was observed. After admission, all of the patients were excluded from hematuric conditions caused by urinary tumors, urinary calculi, infections, trauma, and abnormal coagulation function by relevant laboratory, or imaging examinations. All the patients were also further diagnosed and treated by urethrocystoscopy. Bladder mucosae, bilateral ureteral openings, posterior urethra, and anterior urethra were observed carefully, and we paid special attention to any suspicious abnormal indications such as bleeding, neoplasms, or abnormal protuberances. We also performed transrectal seminal vesicle massage to observe the color of the seminal vesicle fluid seeping from the bilateral ejaculatory orifices. Most of the patients had obvious hemangioma-like protuberances at the 6:00 position between the verumontanum and the external urethral sphincter in lithotomy position, which was significantly different from the surrounding normal urethral mucosa. A few patients had a circular, abnormal vascular protuberance in this area, and some individuals had localized abnormal hemangioma-like protuberances in the anterior urethral area. Electroresection either alone or along with electrocautery (using an SM10 bipolar plasma system at 100 W of output power for electroresection and 80 W for electrocautery) to the abnormal blood vessels or hemangioma-like protuberances were performed carefully and precisely. The range of resection was slightly more than the edge of the lesion by approximately 2 mm, and the depth was usually 4–5 mm (equivalent to the depth of an electric cutting loop). The bottom was usually fulgurated at the same time to destroy the abnormal blood vessels at the edge of the lesion. Foley urethral catheters were routinely retained for 1 or 2 days after the operation.

We used SPSS 18.0 statistical software for all of the statistical analyses. Measurement data that conformed to a normal distribution are expressed by x ± s, whereas non-normally distributed measurement data are expressed by medians and range.

## Results

The age range of the patients was 18–61 years (mean age was 44.1 ± 10.2 years). The duration of symptoms ranged from 1 month to 20 years, and the median symptom duration was 24 months. Of these, 15 cases had a history of less than 1 year, 24 cases of more than 1 year, and 9 cases of more than 5 years. According to the age at onset, there were 5 patients aged less than 30 years, 13 patients aged 30–39 years, 12 patients aged 40–49, and 9 patients aged 50–59 years.

All of the patients showed typical recurrent or persistent hematospermia accompanied by hematuria on the first urination after sexual intercourse or even presented a large amount of fresh blood or blood clots from the urethral orifice after sexual intercourse or masturbation. The amount of blood in the urine was usually significantly more than that in the semen, and the hematuria then disappeared on the second and later urinations after sexual intercourse. The overall characteristic symptoms of the 39 patients are shown in Table 1.

**Table 1:** Symptoms of 39 patients with hematospermia and postcoital hematuria

Symptoms	Number of patients (%)
Hematuria after sexual intercourse	39 (100)
Hematuria with blood clots after sexual intercourse	31 (79.5)
Hematuria after sexual excitement or erection without ejaculation	19 (48.7)
Dysuria with repeated urinary retention and massive hematuria after sexual intercourse	1 (2.6)
Blood dripping from urethra with hematuria after sexual intercourse	2 (5.1)

**Fig. 1:**
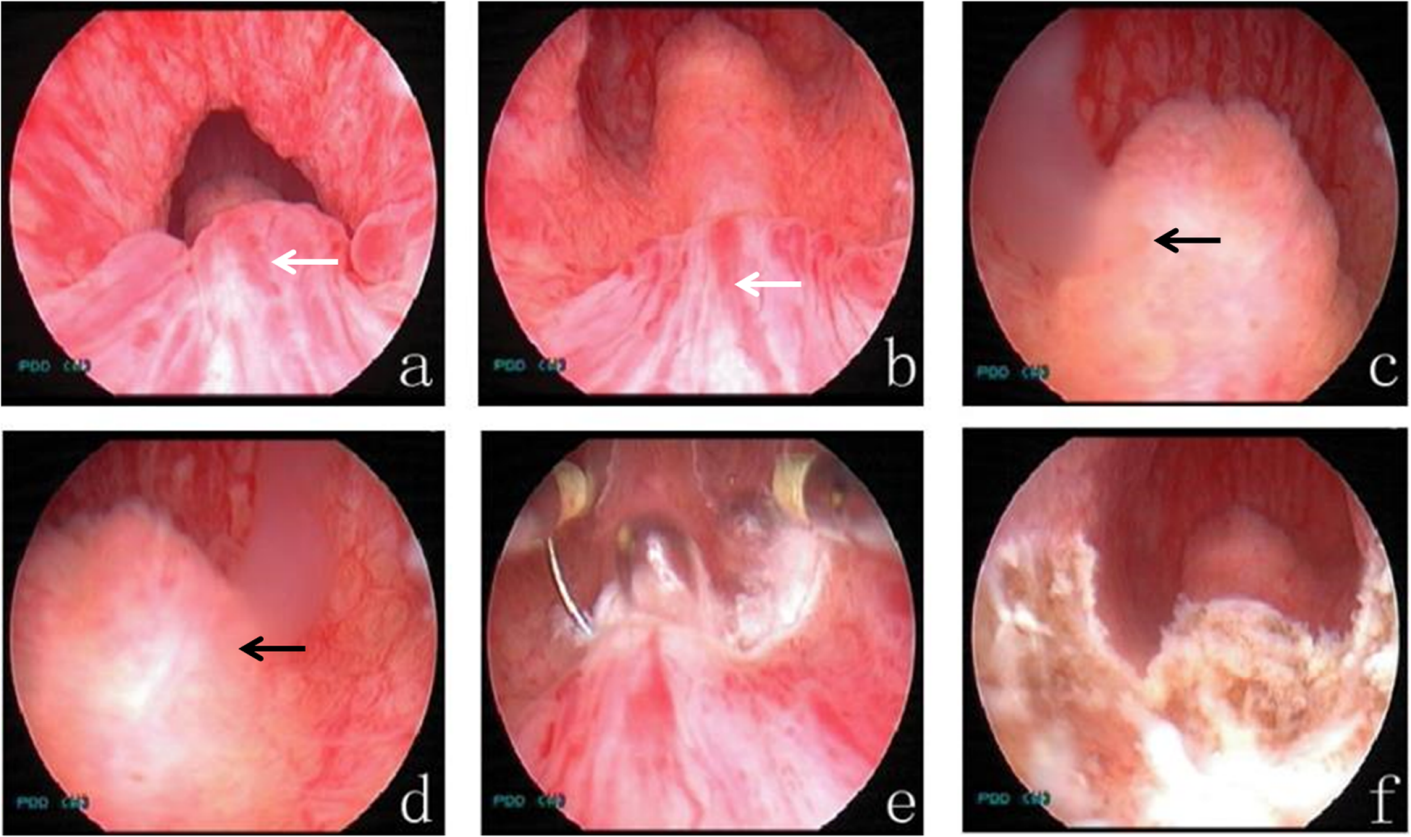
Typical case 1. Endoscopic observation and management of PUH. **a**, **b** There is a dark red hemangioma-like mass located at 6:00 in the lithotomy position, between the verumontanum and the external urethral sphincter, and 0.5–1 cm from the distal part of the verumontanum (white arrow). **c**, **d** There is seepage of milky white, jelly-like seminal vesicle fluid from the orifices of the bilateral ejaculatory ducts upon transrectal seminal vesicle massage, but there was no bleeding in the seminal tract (black arrow). **e**, **f** The PUH was treated by electroscission either alone or electrocautery

**Fig. 2:**
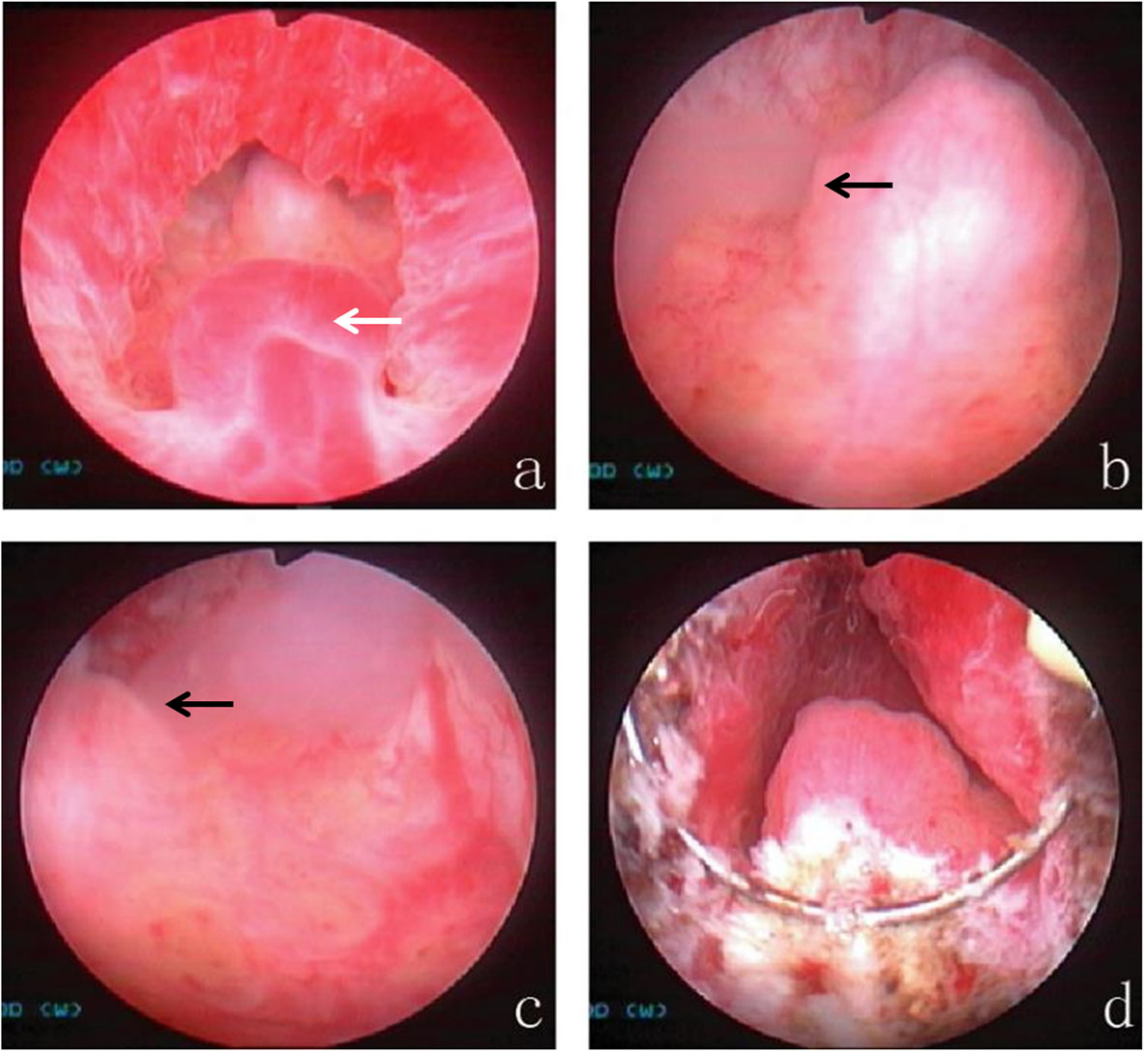
Typical case 2. Endoscopic observation and management of PUH. **a** Typical dark red, hemangioma-like bulge observed 0.5–1 cm from the distal part of the verumontanum, between the verumontanum and the external urethral sphincter (white arrow). **b**, **c** There is seepage of milky white, jelly-like seminal vesicle fluid from the orifices of the bilateral ejaculatory ducts upon transrectal seminal vesicle massage but no bleeding in the seminal tract (black arrow). **d** The PUH was treated by electroscission either alone or along with electrocautery

All of the patients underwent ultrasonography of the urinary system (and CT examination if necessary), and we excluded the lesions caused by tumors, stones, or other common causes of urinary system bleeding. Pelvic magnetic resonance imaging (MRI) was usually performed before surgery, and the results showed that the size and shape of the seminal vesicles were normal and without signs of bleeding. Via urethrocystoscopy, we initially excluded hematuria from the bladder and ureters, and then transrectal seminal vesicle massage further confirmed that there was no bleeding in the seminal vesicles. The typical manifestation we found was a 0.5–0.8 cm × 1.0 cm dark red hemangioma-like mass located at 6:00 in lithotomy position between the verumontanum and the external urethral sphincter, approximately 0.5–1 cm from the distal part of the verumontanum, which was significantly different from the surrounding normal urethral mucosa and prone to bleeding (34/39) (Figs. 1, 2). We observed that some patients had obviously active episodes of hemorrhaging. Lesion localization and patterns in the 39 patients are summarized in Table 2.

**Table 2:** Localization of lesions in the 39 patients as confirmed by urethrocystoscopy

Lesion location	Number of patients (%)
Hemangioma-like mass between the verumontanum and the external urethral sphincter in the 6:00 lithotomy position	34 (87.2)
Abnormal blood vessels with obvious varicosities around the prostatic urethra and verumontanum	3 (7.7)
Circumferential abnormal varicose blood vessels in the plane of the external urethral sphincter	1 (2.6)
Hemangioma-like changes in the bulbourethral area	1 (2.6)

We treated all the lesions with electrocautery alone or along with electroresection. Twenty-one cases underwent direct fulguration at the lesion site because of the small size of the hemangioma or the presence of only varicose blood vessels, and no pathologic specimens were obtained. Eighteen patients underwent electrosurgical excision of abnormal hemangioma-like tissue at the lesion site, and postoperative pathologic findings were cavernous hemangiomas in 15 cases, racemose hemangioma in 1 case, and unclassified hemangiomas in 2 cases.

Each patient was followed up from 3 to 56 months, with a median follow-up period of 16 months. Early postoperative hematuria or urethral bleeding usually disappeared within 2–6 weeks. Except for one case of unilateral acute epididymitis in the early stages after the operation, we uncovered no other related complications—including urethral stricture, dysuria, incontinence, or change in sexual function—in any other patient. The symptoms of hematospermia and hematuria completely disappeared in 37 of the 39 patients during the entire follow-up period, whereas 2 out of the 39 had a recurrence of hematospermia with hematuria 2–3 months after the operation. When re-observed by urethrocystoscopy, the original hemangioma at the 6:00 position disappeared. One patient showed a newly formed circumferential varicosity or hemangioma-like change outside the 6:00 position, whereas the other had hemangioma-like changes in the distal area of the original lesion at the 6:00 position. These patients’ symptoms, however, disappeared after re-electrocautery.

## Discussion

Patients with recurrent or intractable hematospermia accompanied by postejaculatory hematuria are seen very rarely in the clinic, and it is usually easy to misdiagnose or mistreat them due to its atypical clinical manifestations [6]. In an etiologic study of hematospermia, Leary and Aguilo in 1974 found that 4.6% of the 174 cases surveyed were due to urethral varices [22], and more recently, Papp reported that 4–7% of 122 cases were caused by urethral varices [23]. However, only a few studies in the literature during the past 30 years (most of which are case reports) have reported the etiology of hematospermia with postejaculatory hematuria [1–21]. Cattolica in 1982 reported on three patients with severe hematospermia that was accompanied by hematuria, clot formation, and occasional urinary retention, and cystourethroscopy confirmed that the causes were abnormal non-variceal blood vessels in the posterior urethra [24]. In 1997, Hayashi and Furuya reported that the lesions in their patients (characterized by hematospermia and massive hematuria after ejaculation) were located close to the distal end of the verumontanum, and these proved to be urethral hemangiomas by histopathology [25, 26]. Our report featured the largest group of patients studied thus far. Previous reports have suggested that most patients presented in their second or third decade of life [27, 28]. Saito reported 20 patients between 38 and 82 years (mean, 63) with 90% of the patients older than 50 [1]. Our results showed that the age of onset in 64.1% (25/39) of the patients was 30–49 years, and in 87.2% (34/39) of the patients was 30–59 years—certainly an elevated incidence. Analysis of patient medical histories showed that 61.5% (24/39) of the patients had a history of more than 1 year with the condition, of which, 23.1% (9/39) had a history of more than 5 years. This indicated that most of these patients were still affected after repeated treatments, and they had failed to obtain early diagnosis and appropriate and effective treatment.

Patient symptoms were characterized by repeated or stubborn hematospermia accompanied by massive hematuria, and the amount of hemorrhage can be much greater than in simple hematospermia. Blood clots are common in hematuria, and individual patients may also have recurrent urinary retention. At the same time, a few patients may manifest obvious hematuria or urethral bleeding, even if they do not ejaculate after erection or from other sexual stimulation, and this may lead to a fear of sexual intercourse. One patient refused to have sex for as long as 1–2 years for fear of bleeding, which caused stress in this individual’s marriage.

We noted that a previous report suggested that transrectal power Doppler ultrasonography was helpful in the diagnosis of hemangioma, as it revealed a strong blood flow site in the urethra near the apex of the prostate [1]. Unfortunately, we did not perform ultrasonographic examination via Doppler of the entire urethral tract in every patient. However, in order to clarify the etiology, patients in this group were carefully examined by urethrocystoscopy, and our observations were focused on the posterior urethral prostatic region, the verumontanum region, and the distal 1–2 cm region of the verumontanum. The possibility of hematospermia originating from the seminal ducts was completely excluded by our MRI examination before the operation and seminal vesicle massage during the operation. Under the pressure of the endoscopy sheath, abnormal blood vessels are sometimes difficult to observe clearly. Cattolica suggested that cystoscopy during penile flaccidity may miss these lesions that enlarge and become more visible during an erection [24]. Saito proposed that endoscopy with reduced irrigation flow immediately after ejaculation will increase the probability of diagnosing these lesions [1]. Our experience is that it is necessary to constantly adjust the angle of the front end of the endoscope, repeatedly moving it forward and backward, and to reduce the flushing pressure to carefully observe the integrity and smoothness of the urethral mucosa in the aforementioned areas. We usually found that there were isolated smooth pink or dark red, vein-like mass protrusions with clear boundaries at the 6:00 position between the distal part of the verumontanum and the external urethral sphincter. A few patients also showed a circular protrusion in this area. In addition to the above typical manifestations, we also had three patients with widespread and abnormal varicose blood vessels around the prostatic urethra, and verumontanum; one patient showed a circular abnormal enlargement of blood vessels on the plane of the external urethral sphincter; and one patient manifested localized hemangioma-like changes in the anterior urethra (bulbar urethra). Because urethral lesions in this type of patients occur in different areas and are of different extent and size, it is necessary to observe the abnormal changes carefully throughout the entire urethra so as to deliver an appropriate diagnosis and treatment.

Usual methods for the endoscopic resection of PUH are electroresection/electrocautery and Holmium laser resection [3, 5, 19, 20]. The former is a simple and effective method with fewer complications and less recurrence. It has also been reported that endoscopic injection of a sclerosing agent, pingyangmycin, is an alternative therapy for the treatment of hemangiomas [6, 29]. In addition, De León and others believe that Holmium laser treatment of prostatic urethral hemangiomas is effective, without complications, and should become one of the preferred methods for the treatment of prostatic urethral hemangiomas [19]. Regardless of the treatment method used, the objective is to improve the therapeutic effect and reduce the recurrence rate and complications. Thirty-nine patients in our group were treated with transurethral electroscission either alone or along with electrocautery, and we observed no complications during the operation. Only one patient developed acute epididymitis early after the operation, and he was successfully treated with active anti-infection therapy. No other complications such as urethral stricture, dysuria, incontinence, or change in sexual function occurred. Only two cases showed recurrence within 2–3 months postoperatively, and all recovered well after a second use of the same method.

It is reported that depending upon the pattern, hemangioma of the urinary tract is classified as cavernous, capillary, venous, or racemose—with cavernous being the most common [21]. In our study, 18 patients received a histopathologic diagnosis—with cavernous hemangioma in 15 cases, racemose hemangioma in one case, and unclassified hemangioma in two cases. The pathologic types of the cases in our group were obviously consistent with a previous report [21].

The etiology and pathogenesis of PUH—which often occurs between the verumontanum and the external urethral sphincter—remain unclear. Some scholars believe that vascular endothelial growth factor is an important influencing factor, whereas others believe that urethral hemangioma is a congenital disease. The latter may originate from residual embryonic angioblasts—which cannot successfully differentiate into normal blood vessels—leading to the formation of hemangiomas [25]. This possibility cannot be ruled out as it may be due to a degenerative process associated with chronic stimuli and atrophy. A few cases may also be associated with some congenital diseases, including Sturge Weber or Klippel-Trenaunay syndrome [17, 30, 31]. Saito suggested that there was a high intraluminal urethral pressure around the external urethral sphincter, and high venous pressure led to the formation of hemangiomas [1]. From our cases and previously reported data [1, 2, 5], we believe that PUH is more common than reported in the published literature. The possibility of urethral hemangiomas should be considered when patients present with hematospermia accompanied by postejaculatory or posterection hematuria.

This study has several limitations: We did not evaluate the imaging pattern of hemangiomas by Doppler ultrasonography, and we included only a limited number of patients because of low disease prevalence. Only one bipolar plasma system for electroresection either alone or along with electrocautery was used, and we did not compare it with any other methods for the treatment of PUH.

## Conclusion

In summary, as an exceedingly rare and easily misdiagnosed clinical symptom, hematospermia accompanied by postejaculatory or posterection hematuria can be diagnosed by its characteristic manifestations. PUH and abnormal blood vessels in the prostate (as confirmed by cystourethroscopy) were common causes of hematospermia and postejaculatory hematuria. We showed that transurethral resection or fulguration of urethral hemangioma or abnormal blood vessels constitutes a minimally invasive, simple, safe, and effective treatment method.

## Data Availability

The data of the current study are available from the corresponding author upon reasonable request.

## Abbreviations

PUH: Posterior urethral hemangioma
MRI: Magnetic resonance imaging
CT: Computerized tomography

## References

[1] Saito S (2008). Posterior urethral hemangioma: one of the unknown causes of hematuria and/or hematospermia. Urology..

[2] Tian L, Han H, Lei HE, Zhang XD (2018). Clinical features of haematospermia associated with seminal vesicle calculi versus posterior urethral haemangioma. Andrologia..

[3] Han H, Zhou XG, Fan DD, Tian L, Zhang XD (2015). An unusual etiology for hematospermia and treatments that were successful. Urology..

[4] Kang DH, Lee JY, Jung DC, Oh YT, Cho ES, Park SY, Lee KS, Cho KS (2018). Tertiary referral hospital experiences of men presenting with painless postcoital gross hematuria and a suggestion for the management algorithm. Urology..

[5] Soleimani MJ, Shadpour P, Mehravaran K, Kashi AH (2017). Laser treatment for urethral hemangiomas: report of three cases. Urol J..

[6] Yong F, Juan L, Jinhuan W, Haohua Y, Wei C, Jiacong M, Junhang L, Wenwei W (2019). Urethral cavernous hemangioma: a highly misdiagnosed disease (a case report of two patients and literature review). BMC Urol..

[7] Mandal S, Nayak P, Purkait S, Das M, Mahalingam R (2019). Rare images of urethral hemangioma and its management. Urology..

[8] Varea-Malo R, Campos-Juanatey F, Portillo Martín JA, Castillo CL (2019). Multiple urethral hemangiomas associated with urethral stricture: an uncommon aetiology for urethral bleeding. Case Rep Urol..

[9] Itesako T, Eura R, Okamoto Y, Tatarano S, Yoshino H, Nishimura H, Yamada Y, Enokida H, Nakagawa M (2018). Oral propranolol in a child with infantile hemangioma of the urethra. Urology..

[10] Hamada A, Hattahara K, Oyama R, Hirayama K, Masui K, Shichiri Y (2017). Urethral hemangioma with repeated urinary retention by posterection hematuria. Hinyokika Kiyo..

[11] Parshad S, Yadav SP, Arora B. Urethral hemangioma. An unusual cause of hematuria. Urol Int. 2001;66(1):43–5. 10.1159/000056564 11150953

[12] Lauvetz RW, Malek RS, Husmann DA. Treatment of extensive urethral hemangioma with KTP/532 laser. Lasers Surg Med. 1996;18(1):92–5. 10.1002/(SICI)1096-9101(1996)18:1<92::AID-LSM12>3.0.CO;2-D 8850471

[13] Singh DV, Mandal AK (2014). Unusual cause of urethral bleeding in an adolescent: a case in dilemma. Int J Adolesc Med Health..

[14] Abbinante M, Crivellaro S, Guaitoli P, Mastrocinque G, Ammirati E, Frea B (2012). Cavernous hemangioma of the spongious body of the urethra: a case report. Urologia..

[15] Serizawa RR, Nørgaard N, Horn T, Vibits H (2011). Hemangioma of the prostate--an unusual cause of lower urinary tract symptoms: case report. BMC Urol..

[16] Noviello C, Cobellis G, Romano M, Amici G, Martino A (2011). Posterior urethral polyp causing haematuria in children. Pediatr Med Chir..

[17] Tepeler A, Yeşilolva Y, Kılınç A, Aktoz T, Onen A (2011). A mild and rare form of Klippel-Trenaunay syndrome presenting with urethral bleeding due to penile hemangioma. Urology..

[18] Efthimiou I, Kavouras D, Vasilakis P, Katsanis S (2009). Hemangioma of penile urethra-treatment with simple transurethral excision: a case report. Cases J..

[19] de León JP, Arce J, Gausa L, Villavicencio H (2008). Hemangioma of the prostatic urethra: holmium laser treatment. Urol Int..

[20] Khaitan A, Hemal AK (2000). Urethral hemangioma: laser treatment. Int Urol Nephrol..

[21] Jahn H, Nissen HM (1991). Haemangioma of the urinary tract: review of the literature. Br J Urol..

[22] Leary FJ, Aguilo JJ (1974). Clinical significance of hematospermia. Mayo Clin Proc.

[23] Papp GK, Kopa Z, Szabo F (2003). Aetiology of haemospermia. Andrologia.

[24] Cattolica EV (1982). Massive hemospermia: a new etiology and simplified treatment. J Urol.

[25] Hayashi T, Igarashi K, Sekine H (1997). Urethral hemangioma: case report. J Urol.

[26] Furuya S, Ogura H, Tanaka Y (1997). Hemangioma of the prostatic urethra: hematospermia and massive postejaculation hematuria with clot formation. Int J Urol.

[27] Manuel ES, Seery WH, Cole AT (1977). Capillary hemangioma of the male urethra: case report with literature review. J Urol..

[28] Roberts JW, Devine CJ (1983). Urethral hemangioma: treatment by total excision and grafting. J Urol..

[29] Bissada NK, Frangos DN, Ferentzi C (1994). Management of extensive urethral hemangiomas with endoscopic sclerotherapy:case report. J Urol..

[30] Klein TW, Kaplan GW (1975). Klippel- Trenaunay syndrome associated with urinary tract hemangiomas. J Urol.

[31] Lei H, Guan X, Han H (2018). Painless urethral bleeding during penile erection in an adult man with Klippel-Trenaunay syndrome: a case report. Sex Med..

